# Эндокринные нарушения после комбинированной химиолучевой терапии у больных лимфомой Ходжкина

**DOI:** 10.14341/probl13124

**Published:** 2023-05-11

**Authors:** М. С. Войтко, В. В. Климонтов, Т. И. Поспелова, Я. Ю. Шебуняева, О. Н. Фазуллина

**Affiliations:** Новосибирский государственный медицинский университет; Городская клиническая больница № 2; Новосибирский государственный медицинский университет; Научно-исследовательский институт клинической и экспериментальной лимфологии — филиал ФГБНУ «Федеральный исследовательский центр Институт цитологии генетики Сибирского отделения Российской академии наук»; Новосибирский государственный медицинский университет; Городская клиническая больница № 2; Новосибирский государственный медицинский университет; Научно-исследовательский институт клинической и экспериментальной лимфологии — филиал ФГБНУ «Федеральный исследовательский центр Институт цитологии генетики Сибирского отделения Российской академии наук»

**Keywords:** лимфома Ходжкина, гипотиреоз, гиперпаратиреоз, гипогонадизм, лучевая терапия, минеральная плотность костной ткани

## Abstract

**ОБОСНОВАНИЕ:**

ОБОСНОВАНИЕ. Лимфома Ходжкина (ЛХ) — одно из наиболее частых злокачественных лимфопролиферативных заболеваний. Полихимиотерапия (ПХТ) и лучевая терапия, используемые в лечении ЛХ, индуцируют многочисленные токсические эффекты, приводящие к функциональным нарушениям эндокринной системы. Гормональные нарушения при ЛХ, их связь с проведенной терапией недостаточно изучены.

**ЦЕЛЬ:**

ЦЕЛЬ. Оценить нарушения функции щитовидной железы, паращитовидных и половых желез у больных ЛХ на этапе клинико-гематологической ремиссии.

**МАТЕРИАЛЫ И МЕТОДЫ:**

МАТЕРИАЛЫ И МЕТОДЫ. Проведен скрининг нарушения функции щитовидной железы, паращитовидных и половых желез у 160 взрослых больных ЛХ (55 мужчин и 105 женщин) на этапе ремиссии, индуцированной ПХТ или комбинированной химиолучевой терапией. Контрольную группу составили 40 здоровых лиц, сопоставимых по возрасту. Уровни тиреотропного гормона, общего трийодтиронина, свободного тироксина, паратиреоидного гормона (ПТГ), фолликулостимулирующего гормона (ФСГ), лютеинизирующего гормона (ЛГ), свободного тестостерона, дегидроэпиандростерона сульфата (ДГЭА-С), глобулина, связывающего половые гормоны (ГСПГ), определяли в сыворотке крови методом иммуноферментного анализа. Исследование минеральной плотности костной ткани (МПКТ) проводили методом двухэнергетической рентгеновской денситометрии.

**РЕЗУЛЬТАТЫ:**

РЕЗУЛЬТАТЫ. Наиболее распространенными эндокринными нарушениями у больных ЛХ были гипотиреоз (25%), гиперпаратиреоз (15,6%) и гипогонадизм (29% мужчин и 25,3% женщин). Гипотиреоз встречался достоверно чаще у больных после химиолучевой терапии, чем у пациентов, получавших только ПХТ (χ2=9,4; р=0,002). У больных с гиперпаратиреозом уровень ПТГ отрицательно коррелировал с МПКТ в поясничном отделе позвоночника (r=-0,74; p=0,00002) и в шейке бедра (r=-0,66; p=0,0003). Мужчины после ЛХ имели достоверно более низкий уровень свободного тестостерона в сравнении с контрольной группой (p=0,04), уровни ЛГ и ФСГ оказались повышены: p=0,0004 и p=0,04 соответственно. Уровень ДГЭА-С у мужчин с ЛХ был снижен (p=0,0009). У 13 (23,6%) мужчин зафиксирована повышенная концентрация ГСПГ. Женщины репродуктивного возраста с ЛХ, в сравнении с женщинами контрольной группы, имели более высокий уровень ЛГ в лютеиновую фазу (p=0,05) и более высокий уровень ФСГ в фолликулиновую фазу (p=0,02).

**ЗАКЛЮЧЕНИЕ:**

ЗАКЛЮЧЕНИЕ. Полученные данные указывают на высокую распространенность дисфункции щитовидной железы, паращитовидных и половых желез у больных ЛХ на этапе ремиссии. Очевидна необходимость скрининга эндокринных нарушений у этой категории больных.

## Abstract (en)

**BACKGROUND:**

BACKGROUND: Hodgkin’s lymphoma (HL) is one of the most common malignant lymphoproliferative diseases. Chemotherapy and radiotherapy used in the treatment of LH induce a number of toxic effects leading to dysfunction of endocrine system. Hormonal disorders in HL and their relationships with the therapy used remain to be clarified.

**AIM:**

AIM: To assess disorders of the endocrine function of thyroid, parathyroid glands and gonads in HL survivors.

**MATERIALS AND METHODS:**

MATERIALS AND METHODS: Screening of endocrine dysfunction of the thyroid, parathyroid glands and gonads was performed in 160 adult patients with HL, 55 men and 105 women, at remission stage induced by chemotherapy or chemoradiotherapy. Forty healthy subjects, matched by age, were acted as control. The levels of TSH, T3, free T4, PTH, FSH, LH, free testosterone, dehydroepiandrosterone sulfate (DHEA-S), and sex-hormone binding globulin (SHBG) were measured in blood serum by ELISA. Bone mineral density (BMD) was assessed by DEXA.

**RESULTS:**

RESULTS: Hypothyroidism (25%), hyperparathyroidism (15.6%) and hypogonadism (29% of men and 25.3% of women) were the most prevalent endocrine disorders in LH survivors. Hypothyroidism was significantly more common in patients after chemoradiotherapy than in those who received only chemotherapy (χ2=9.4, р=0.002). In patients with hyperparathyroidism, there were negative correlations between PTH levels and BMD in the lumbar spine (r=-0.74, p=0.00002) and in the femoral neck (r=-0.66, p=0.0003). Men with HL demonstrated lower free testosterone concentrations when compared to control (p=0.04); LH and FSH levels were elevated (p=0.0004 and p=0.04, respectively). In men with HL the levels of DHEA-S were reduced (p=0.0009). The increased SHBG concentrations were revealed in 13 (23.6%) men. Women of reproductive age with HL had higher levels of LH in the luteal phase (p=0.05) and FSH in the follicular phase (p=0.02) than controls.

**CONCLUSION:**

CONCLUSION: The data indicate a high prevalence of the dysfunctions of thyroid, parathyroid glands, and gonads in HL survivors. Screening for endocrine disorders in these patients is highly recommended.

## ОБОСНОВАНИЕ

Лимфома Ходжкина (ЛХ) — одно из наиболее частых злокачественных лимфопролиферативных заболеваний среди лиц трудоспособного возраста. Заболеваемость ЛХ составляет 2,1–2,8 случая на 100 000 населения в год [1]. Распределение больных ЛХ по возрасту имеет бимодальный характер: основной пик заболеваемости приходится на возраст от 15 до 35 лет, второй пик наблюдается после 50 лет [2]. В последние десятилетия достигнуто значительное улучшение результатов лечения ЛХ: в настоящее время излечиваются более 80% пациентов [3]. Учитывая высокую выживаемость и молодой возраст основной группы пациентов с ЛХ, особую актуальность приобретает вопрос о реабилитации и качестве жизни этих больных. Полихимиотерапия (ПХТ) и лучевая терапия (ЛТ), используемые в лечении ЛХ, индуцируют многочисленные токсические эффекты, лежащие в основе патогенеза отдаленных последствий лечения [4]. К числу последних относятся и поражения эндокринной системы. Известно, что цитостатические препараты и радиация могут оказывать повреждающий эффект на клетки гипофиза, щитовидной железы (ЩЖ), гонад и других эндокринных органов [5]. Вместе с тем структура поражений эндокринной системы у больных ЛХ в отдаленный период клинико-гематологической ремиссии, особенности их клинического течения и связь с применявшейся терапией изучены недостаточно.

## ЦЕЛЬ ИССЛЕДОВАНИЯ

Оценить распространенность и факторы риска нарушений функции эндокринных желез у больных ЛХ, перенесших ПХТ и ЛТ, на этапе клинико-гематологической ремиссии.

## МАТЕРИАЛЫ И МЕТОДЫ

Место и время проведения исследования

Место проведения. Набор пациентов проводился на базе гематологического отделения ГБУЗ НСО «Городская клиническая больница №2» г. Новосибирска. Уровень гормонов определяли с помощью иммуноферментного анализа (ИФА) в центральной исследовательской лаборатории НГМУ Минздрава России. Двухэнергетическую рентгеновскую абсорбциометрию (DXA) проводили в клинике НИИКЭЛ — филиале ИЦиГ СО РАН (г. Новосибирск, Россия).

Время исследования. Исследование проводилось в период с декабря 2018 г. по апрель 2020 г.

Изучаемые популяции

В исследовании приняли участие 160 больных ЛХ, находящихся в клинико-гематологической ремиссии после перенесенной химиолучевой терапии. В контрольную группу вошли 40 человек, сопоставимых по возрасту и полу, без тяжелых хронических заболеваний.

Критерии включения для основной группы: гистологически верифицированный диагноз ЛХ; возраст от 18 до 65 лет; наличие у пациента клинико-гематологической ремиссии, индуцированной ПХТ и ЛТ; подписанное информированное согласие на участие в исследовании.

Критерии исключения: заболевания эндокринной системы (гипо- и гипертиреоз, гипо- и гиперкортицизм, гипогонадизм, ожирение 3-й степени), возникшие до постановки диагноза ЛХ; сопутствующие онкологические заболевания; печеночная недостаточность; терминальная стадия хронической болезни почек; застойная сердечная недостаточность; ВИЧ-инфекция.

При формировании контрольной группы применяли те же критерии исключения, что и в основной группе.

Способ формирования выборки из изучаемой популяции

Выборка пациентов с ЛХ была сформирована согласно данным госпитального регистра и амбулаторных карт гематологического отделения ГБУЗ НСО «Городская клиническая больница №2» г. Новосибирска.

Дизайн исследования

Одноцентровое обсервационное одномоментное (поперечное) неинтервенционное исследование.

Методы

Оценка эффективности терапии у больных ЛХ проводилась с помощью компьютерной томографии шеи, грудной клетки, органов брюшной полости и малого таза, ультразвукового исследования (УЗИ) органов брюшной полости; у части пациентов выполнена позитронно-эмиссионная томография всего тела с 18F-фтордезоксиглюкозой. Комплекс клинического обследования, кроме того, включал исследование гемограммы, биохимический анализ крови с определением липидного спектра, общего белка, трансаминаз, креатинина, общего кальция, фосфора. Исследование спектра гормонов в сыворотке крови проводили методом ИФА на анализаторе Multiskan FC (Thermo Fisher Scientific Instruments Co., China). Уровень тиреотропного гормона (ТТГ), свободного тироксина (Т4св), общего трийодтиронина (Т3), лютеинизирующего гормона (ЛГ), фолликулостимулирующего гормона (ФСГ) определяли с помощью тест-систем фирмы «Вектор-Бест» (Россия), уровень паратиреоидного гормона (ПТГ) — с помощью наборов DiaSource (Бельгия). У мужчин, кроме того, измеряли концентрации свободного тестостерона с помощью тест-систем DRG (Германия), дегидроэпиандростерона сульфата (ДГЭА-С) и глобулина, связывающего половые гормоны (ГСПГ), с помощью тест-систем Diagnostics Biochem Canada Inc (Канада). У больных с отклонениями в уровне ТТГ и/или тиреоидных гормонов выполняли УЗИ ЩЖ, исследование антител к тиреопероксидазе. У пациентов с изменениями уровня паратгормона, показателей кальциево-фосфорного обмена проводили исследование МПКТ в поясничном отделе позвоночника, проксимальном отделе бедренной кости и предплечье недоминантной руки методом DXA на денситометре Lunar Prodigy (GE, США).

Статистический анализ

Статистическая обработка проведена с использованием программы STATISTICA 10 (StatSoft Inc, 2011, CША). Гипотезу о нормальности распределения проверяли с помощью критерия Колмогорова–Смирнова. Учитывая, что распределение большинства изученных признаков было отличным от нормального, применяли методы непараметрической статистики. Проверку гипотезы о равенстве генеральных средних в сравниваемых группах проводили с помощью непараметрического критерия Манна–Уитни. Для оценки статистической значимости различий двух или нескольких относительных показателей применяли критерий χ2 Пирсона. Для всех видов анализа различия считали достоверными при p<0,05. Данные представлены как медианы, 25 и 75 перцентили, или наименьшие и наибольшие значения.

Этическая экспертиза

Протокол исследования одобрен локальным этическим комитетом ФГБОУ ВО «Новосибирский государственный медицинский университет» Минздрава РФ (протокол №111 от 29.11.2018).

## РЕЗУЛЬТАТЫ

В исследование включены 160 больных, 55 мужчин и 105 женщин (42 женщины в постменопаузе). Медиана возраста составила 42 года [ 25, 75 перцентиль: 33 и 55 лет]. В обследуемой группе больных преобладали пациенты с распространенными стадиями (III–IV) заболевания по классификации Ann Arbor в дополнении Cotswolds 1988 г. (n=89), I стадия ЛХ диагностирована у 2 человек, II — у 69. В соответствии с гистологической классификацией ЛХ наиболее часто диагностировали нодулярный склероз и смешанно-клеточный вариант — у 72 (45%) и 73 (45,6%) пациентов соответственно; лимфоидное истощение диагностировано у 5 пациентов, вариант, богатый лимфоцитами, — у 3, у 7 больных выявлена нодулярная ЛХ с лимфоидным преобладанием.

В качестве индукции ремиссии все пациенты получили ПХТ I линии. Количество циклов ПХТ составляло 2–12 (в среднем 6). Лечение по программе ABVD (адриамицин 25 мг/м2, блеомицин 10 мг/м2, винбластин 6 мг/м2, дакарбазин 375 мг/м2, все препараты вводятся в 1-й и 15-й дни с интервалом 2 нед) получили 63 пациента. Комбинация схем ABVD и BEACOPP (циклофосфан 650 мг/м2 в 1-й  день, адрибластин 25 мг/м2 в 1-й день, вепезид по 100 мг/м2 в 1–3-й дни, прокарбазин по 100 мг/м2 в 1–7-й дни, преднизолон по 40 мг/м2 в 1–14-й дни, блеомицин 10 мг/м2 в 8-й день и винкристин 1,4 мг/м2 в 8-й день, курс возобновляется на 22-й день) использована у 97 больных. Последующая ЛТ на область шейно-надключичных лимфатических узлов назначалась 95 пациентам: у 84 из них суммарная очаговая доза (СОД) составила 30 Гр, у 11 — 36 Гр. Восемь больных получили ЛТ на шейно-надключичные и паховые лимфоузлы одновременно, СОД составила 36 Гр у 3 больных, 30 Гр — у 5. Курс ПХТ II линии проведен у 41 пациента. Трансплантация аутологичных гемопоэтических стволовых клеток выполнена у 5 больных. Длительность наблюдения после ПХТ и ЛТ составила от 1 до 28 лет (медиана — 6,5 года).

В контрольную группу вошли 40 лиц без тяжелых хронических заболеваний, 24 женщины (включая 6 женщин в постменопаузе) и 16 мужчин, от 18 до 58 лет (медиана — 40 лет).

Уровни гормонов ЩЖ у обследованных больных, получавших ПХТ или комбинированное химиолучевое лечение, представлены в табл. 1. Медиана уровня ТТГ у больных ЛХ составил 2,25 (1,1; 6,4) мМЕ/л. Отклонения показателей от референсных значений были выявлены у 40 (25%) пациентов. Повышение уровня ТТГ и снижение уровня Т4св и/или Т3 выявлены у 12 больных, изолированное повышение ТТГ без изменения уровня периферических гормонов — у 18. Данные изменения встречались у 24 женщин и 6 мужчин. У 10 пациентов, 9 женщин и 1 мужчины, выявлено снижение уровня периферических гормонов ЩЖ при нормальном или сниженном уровне ТТГ, что с определенной долей вероятности может свидетельствовать о синдроме эутиреоидной патологии (СЭП), но не исключает наличия центрального гипотиреоза.

**Table:** Таблица 1. Уровни ТТГ и гормонов щитовидной железы в сыворотке крови у больных ЛХ в периоде ремиссии в зависимости от метода лечения

Параметр	ПХТ и ЛТ в анамнезе (n=95), p1	ПХТ в анамнезе (n=65), p2	Контрольная группа (n=40), pк	р
ТТГ, мМЕ/л	2,1 (1,1; 4,05)	1,35 (0,9; 2,1)	1,05 (0,8; 1,55)	p1–2=0,006 p1–к=0,0003 p2–к=0,007
Т4св, пмоль/л	11,3 (10,4; 13,5)	11,8 (10,7; 13,7)	12,9 (11,4; 15,4)	p1–2=0,39 p1–к=0,007 p2–к=0,01
Т3, нмоль/л	1,4 (1,3; 1,8)	1,6 (1,3; 1,8)	1,7 (1,4; 2,2)	p1–2=0,03 p1–к=0,08 p2–к=0,17

У больных, находящихся в ремиссии после комбинированного химиолучевого лечения, дисфункция ЩЖ встречалась достоверно чаще, чем у пациентов, получавших только ПХТ (32 и 8 человек соответственно; 33,7 и 12,3%; χ2=9,4; р=0,002). Среди 95 больных с облучением шейно-надключичных областей в анамнезе распространенность повышения ТТГ зависела от дозы радиации: 22 (26,1%) при СОД 30 Гр, 8 (72,7%) при СОД 36 Гр (χ2=9,75; р=0,002).

Клиническая картина у пациентов с манифестным гипотиреозом (n=12) была представлена отечным синдромом (n=6), диспепсическим синдромом (запоры, метеоризм и др., n=5), дислипидемией (n=10), нормохромной нормоцитарной анемией (n=4), у 4 женщин репродуктивного возраста отмечены нарушения цикла по типу олигоопсоменореи. Все больные данной группы имели избыточную массу тела или ожирение (медиана индекса массы тела 27,7 кг/м2). У трех больных при дальнейшем обследовании выявлен диффузно-узловой нетоксический зоб, у одного верифицирован аутоиммунный тиреоидит.

Медиана паратгормона у больных ЛХ оказалась достоверно выше, чем у лиц контрольной группы: 67,8 (38,6; 102,8) и 47,1 (33,5; 55,7) пг/мл соответственно, р<0,001. У 25 (15,6%) пациентов уровень ПТГ свидетельствовал о вероятности вторичного гиперпаратиреоза. Уровни кальция и фосфора в данной группе пациентов в большинстве случаев (n=21) не выходили за пределы референсного интервала. Медиана уровня общего кальция составила 2,43 (2,38; 2,52) ммоль/л, медиана уровня фосфора — 1,26 (0,98; 1,4) ммоль/л. У 4 больных отмечалось умеренное повышение уровня сывороточного кальция в диапазоне от 2,62–2,64 ммоль/л, уровень фосфора у этих больных был от 0,78 до 0,82 ммоль/л. У 2 пациентов с высокой концентрацией ПТГ выявлен хронический калькулезный холецистит, у 1 — мочекаменная болезнь. У 16 больных с повышенным уровнем ПТГ зафиксировано снижение МПКТ: остеопения (n=7) или остеопороз (n=9). Показатели МПКТ в поясничном отделе позвоночника составили 1,096 (0,972; 1,308) г/см2, в шейке бедра — 0,964 (0,819; 1,03) г/см2, в предплечье недоминантной руки — 0,695 (0,631; 0,81) г/см2. Повышенный уровень ПТГ отрицательно коррелировал с МПКТ в поясничном отделе позвоночника (r=-0,74; p=0,00002) и в шейке бедра (r=-0,66; p=0,0003). Функция почек (уровень креатинина и скорость клубочковой фильтрации) не отличалась у больных с нормальным и повышенным уровнем ПТГ.

У 3 больных диагностировано снижение уровня ПТГ, у 2 из них имелись клинические признаки вероятного гипопаратиреоза (парестезии, фибриллярные подергивания мышц), уровень кальция находился в диапазоне от 1,9 до 2,2 ммоль/л. Все 3 пациента получали ПХТ по протоколу BEACOPP, 2 из них была назначена ЛТ в СОД 30 Гр на шейно-надключичную область. У 1 пациента также был диагностирован субклинический гипотиреоз.

Нами не отмечено различий в уровне ПТГ и показателях фосфорно-кальциевого обмена между пациентами, получавшими ПХТ и химиолучевое лечение (табл. 2).

**Table:** Таблица 2. Уровень ПТГ и показатели кальциево-фосфорного обмена у больных ЛХ в периоде ремиссии в зависимости от метода лечения

Параметр	ПХТ и ЛТ в анамнезе (n=95), p1	ПХТ в анамнезе (n=65), p2	Контрольная группа (n=40), pк	р
ПТГ, пмоль/л	83,3 (49,6; 101)	96 (39,6; 101)	47,1 (33,5; 55,7)	p1–2=0,96 p1–к =0,000001 p2–к=0,000008
Кальций общий, ммоль/л	2,45 (2,38; 2;61)	2,41 (2,38; 2,5)	2,39 (2,26; 2,48)	p1–2=0,35 p1–к=0,07 p2–к=0,25
Фосфор, ммоль/л	1,25 (0,82; 1,4)	1,28 (1; 1,4)	1,22 (1; 1,4)	p1–2=0,27 p1–к=0,34 p2–к=0,87

Мужчины с ЛХ имели достоверно более низкий уровень свободного тестостерона в сравнении с контрольной группой: 7,7 (4,1; 12,9) и 11,7 (8,1; 15,3) нмоль/л соответственно (p=0,04). Уровни ЛГ и ФСГ у больных ЛХ, напротив, оказались повышены: 6,8 (5,5; 8,5) и 4,2 (3,35; 5,9) мМЕ/мл (p=0,0004), 5,3 (4,3; 8,8) и 4,6 (3,6; 5,4) мМЕ/мл соответственно (p=0,04). Снижение уровня свободного тестостерона до значений, указывающих на гипогонадизм (менее 12,1 нмоль/л), выявлено у 16 из 55 обследованных мужчин с ЛХ (29%), в то время как в группе контроля — у 3. У большинства этих пациентов гипогонадизм был нормогонадотропным; повышение ЛГ зафиксировано лишь у 1 больного, повышение ФСГ — у 5. Все пациенты с гипергонадотропным гипогонадизмом получили ПХТ по протоколам BEACOPP в эскалированных дозах, 3 больным проведена II линия ПХТ по протоколу DHAP.

Медиана ДГЭА-С у мужчин с ЛХ была достоверно ниже, чем в контрольной группе: 1,38 (0,9; 1,9) и 1,92 (1,76; 2,35) мкг/мл соответственно (p=0,0009). Снижение уровня ДГЭА-С выявлено у пяти мужчин (9%); все эти пациенты имели изолированное (без повышения уровня гонадотропинов) снижение концентрации свободного тестостерона. Установлено, что все мужчины с дефицитом ДГЭА-С получали противоопухолевую терапию по протоколам, содержащим алкилирующие цитостатики: ABVD, BEACOPP-14, BEACOPP esc. У 13 (23,6%) мужчин была повышена концентрация ГСПГ, среди них у 10 отмечалось снижение уровня свободного тестостерона, у одного — в сочетании с увеличением ЛГ. У 5 пациентов с повышением уровня ГСПГ диагностировано повышение трансаминаз в крови (медиана уровня АЛТ 48 (43; 55) Ед/л, АСТ — 46,5 (44; 49) Ед/л), что, вероятно, связано с гепатотоксичностью, индуцированной ПХТ по протоколу BEACOPP.

Исследование у женщин проводилось на различных этапах менструального цикла, поэтому сравнение гормонов репродуктивной системы в фолликулиновую, лютеиновую, овуляторную фазы менструального цикла, а также в постменопаузе проводилось отдельно. Уровень гонадотропных гормонов у женщин с ЛХ колебался в широком диапазоне: ФСГ от 0,3 до 107 мМЕ/мл, ЛГ от 0,3 до 62,3 мМЕ/мл. При этом женщины репродуктивного возраста с ЛХ, в сравнении с женщинами контрольной группы, имели более высокий уровень ЛГ в лютеиновую фазу: 8,9 (4,8; 13,2) и 6,8 (5,1; 11,4) мМЕ/мл, p=0,05, и более высокий уровень ФСГ в фолликулиновую фазу: 5,5 (3,4; 8,2) и 2,5 (2,2; 3,4) мМЕ/мл, p=0,02. У пациенток с ЛХ, находящихся в постменопаузе, значимых различий с контролем в уровнях ФСГ и ЛГ не зафиксировано.

Клинические проявления дисфункции яичников выявлены у 16 (25,3%) из 63 женщин репродуктивного возраста. Из них у 9 женщин имела место аменорея, у 7 — олигоопсоменорея. Дисфункция яичников с преобладанием высоких значений гонадотропных гормонов верифицирована у 14 женщин; 5 из них проводилась лучевая терапия на область пахово-подвздошных лимфатических узлов (СОД 36 Гр у 3, СОД 30 Гр у 2). Гипогонадотропный гипогонадизм (со снижением уровня ЛГ и ФСГ) зафиксирован в двух случаях. Следует отметить, что у пациенток, получавших ПХТ по протоколам BEACOPP-14, BEACOPP esc, достоверно чаще диагностировали нарушения со стороны репродуктивной системы, чем у женщин, получавших терапию только по схеме ABVD (χ2=6,0; p=0,02).

## ОБСУЖДЕНИЕ

Репрезентативность выборок

Учитывая достаточный объем выборки и ограниченный набор критериев невключения, можно полагать, что выборка пациентов, включенных в исследование, достаточно репрезентативна по отношению к пациентам с ЛХ, перенесшим ПХТ или химиолучевое лечение и находящимся в клинико-гематологической ремиссии.

Сопоставление с другими публикациями

В данной работе показана высокая распространенность клинических признаков и гормональных изменений, которые могут свидетельствовать о нарушении функции эндокринных желез у больных с ЛХ в состоянии клинико-гематологической ремиссии. У 25% обследованных пациентов выявлены признаки вероятного гипотиреоза. Сведения о распространенности гипотиреоза, развивающегося после химиолучевой терапии у больных ЛХ, сильно отличаются в зависимости от численности групп пациентов, длительности периода наблюдения, соотношения больных, получивших только ПХТ, лучевое или комбинированное химиолучевое лечение, и диапазона доз облучения [6][7]. Считается, что у больных ЛХ нарушение функции ЩЖ при проведении ЛТ на область шеи и верхнего средостения является следствием прямого лучевого повреждения эндотелия сосудов и паренхимы ЩЖ [8]. С этим согласуются полученные нами данные о более высоком риске развития гипотиреоза у пациентов, получавших химиолучевое лечение, и связи риска гипотиреоза с СОД. Другие исследователи также отмечают, что именно СОД в первую очередь ассоциирована с появлением функциональных изменений ЩЖ у больных ЛХ [9]. Кроме того, ряд авторов указывают на то, что терапия противоопухолевыми агентами может приводить к дисфункции ЩЖ [10]. Возможными факторами риска функциональных изменений ЩЖ у больных ЛХ являются также возраст на момент химиолучевой терапии, длительность наблюдения и женский пол [11].

Вместе с тем у 10 пациентов с ЛХ зафиксировано снижение уровня гормонов ЩЖ при нормальной или сниженной концентрации ТТГ, что может указывать на наличие СЭП. Так, при тяжелой соматической патологии, в том числе и у лиц со злокачественными новообразованиями без сопутствующих заболеваний ЩЖ, могут выявляться изменения уровней тиреоидных гормонов. В основе патогенеза СЭП лежит нарушение дейодирования Т3 в печени, увеличение или уменьшение связывания гормонов ЩЖ с белками плазмы и нарушение секреции ТТГ [12]. Выявленный дефицит тиреоидных гормонов на фоне нормального или сниженного уровня ТТГ не позволяет достоверно судить о развитии вторичного гипотиреоза в результате поражения гипофиза противоопухолевой терапией. Выявленные изменения требуют дальнейшего наблюдения.

Исследование функции паращитовидных желез показало наличие признаков, которые могут свидетельствовать о гиперпаратиреозе, у 15,6% больных. Гиперфункция паращитовидных желез в большинстве случаев не сопровождалась изменением показателей фосфорно-кальциевого обмена, что, вероятно, указывает на ее вторичный характер, хотя и не позволяет исключить нормокальциемический вариант первичного гиперпаратиреоза. Вероятными причинами повышения уровня ПТГ у пациентов с ЛХ являются прямое воздействие ЛТ на щитовидную и паращитовидные железы с длительным латентным периодом, нарушения всасывания кальция в кишечнике, дефицит витамина D, а также прием тиазидовых диуретиков для предотвращения волемической перегрузки у больных ЛХ [13]. У 4 больных повышение ПТГ сопровождалось умеренной гиперкальциемией, что потребовало исключения первичного гиперпаратиреоза. При проведении дифференциальной диагностики следует учитывать, что причиной увеличения уровня сывороточного кальция у больных ЛХ может быть локальная активация процессов костной резорбции в связи с выработкой опухолевыми клетками ФНО-альфа и интерлейкина-1 или гуморальных активаторов остеокластов, в частности ПТГ-подобного пептида. Кроме того, гиперкальциемия при злокачественных опухолях также может быть вызвана чрезмерной выработкой кальцитриола. Опухолевые клетки или окружающие лимфоциты сверхэкспрессируют 1α-гидроксилазу, что вызывает эктопическое превращение 25-гидроксивитамина D в 1,25-дигидроксивитамин D [14]. У большинства пациентов с повышенным уровнем ПТГ нами зафиксированы остеопения или остеопороз, при этом прослеживались отрицательные корреляции между ПТГ и МПКТ в позвоночнике и шейке бедра. Таким образом, вторичный гиперпаратиреоз может вносить вклад в генез остеопороза у больных ЛХ.

Снижение уровня ПТГ в нашей выборке пациентов зафиксировано в 3 случаях (1,8%). Известно, что дефицит ПТГ у онкологических больных может быть следствием гипомагниемии [15], одной из причин которой является прием химиотерапевтических препаратов, таких как цисплатин, винкристин и вепезид (входят в состав протоколов BEACOPP и DHAP для лечения ЛХ) [16]. Внешнее облучение шеи также рассматривается как этиологический фактор снижения ПТГ [17]. При этом считается, что лучевое поражение паращитовидных желез у больных ЛХ встречается гораздо реже, чем поражение ЩЖ [18].

Поражение репродуктивной системы — один из наиболее известных побочных эффектов ПХТ. Данные о частоте гипогонадизма у больных ЛХ весьма вариабельны [19]. В нашем исследовании клинические и лабораторные проявления дисфункции яичников выявлены у 25,3% женщин репродуктивного возраста, снижение уровня свободного тестостерона зафиксировано у 29% обследованных мужчин. Возникновение гонадотоксичности главным образом ассоциируется с влиянием алкилирующих цитостатических препаратов, используемых в большинстве схем химиотерапии. Выраженность гонадотоксического эффекта может различаться в зависимости от суммарной дозы препарата или комбинаций алкилирующих препаратов с другими цитостатическими препаратами в протоколах ПХТ [20].

В данном исследовании нами выявлены некоторые особенности гипогонадизма у мужчин с ЛХ. В большинстве случаев гипогонадизм является нормогонадотропным, у части больных дефицит тестостерона был ассоциирован со снижением уровня ДГЭА-С и с повышением концентрации ГСПГ. Одновременный дефицит тестостерона и надпочечникового андрогена ДГЭА-С у мужчин с ЛХ в периоде ремиссии способствует развитию эректильной дисфункции, абдоминального ожирения, снижению мышечной массы и МПКТ; таким образом, эти гормональные изменения могут ухудшать качество жизни и прогноз пациентов. В патогенезе гипогонадизма у части пациентов с ЛХ играет роль повышение уровня ГСПГ. Основным местом синтеза данного белка-переносчика является печень, уровень ГСПГ может возрастать при поражениях этого органа: гепатоцеллюлярной карциноме, неалкогольной жировой болезни печени. По нашим данным, уровень ГСПГ целесообразно оценивать, в частности, у больных с ЛХ и гепатотоксичностью, индуцированной ПХТ.

Клиническая значимость результатов

Проведенное исследование демонстрирует высокую распространенность эндокринных нарушений у пациентов с ЛХ, перенесших ПХТ и ЛТ и находящихся в состоянии клинико-гематологической ремиссии. Очевидна необходимость участия специалистов-эндокринологов в диспансерном наблюдении данной категории больных.

Ограничения исследования

Ограничениями исследования являются одномоментный дизайн, не позволяющий судить о причинно-следственных связях между признаками, а также набор больных в одном клиническом центре. В части оценки функции эндокринной системы исследование является скрининговым, выявленные гормональные изменения требуют верификации при динамическом наблюдении.

Направления дальнейших исследований

Полученные результаты указывают на необходимость дальнейшего изучения отдаленных последствий химиолучевой терапии у больных ЛХ, а также разработки программ скрининга и коррекции эндокринных нарушений у данной категории пациентов.

## ЗАКЛЮЧЕНИЕ

Проведенное скрининговое исследование свидетельствует о достаточно высокой распространенности эндокринной дисфункции у больных ЛХ, находящихся в периоде клинико-гематологической ремиссии после ПХТ и химиолучевой терапии. Данные изменения указывают на высокий риск развития гипотиреоза (у 25% больных), гиперпаратиреоза (у 15,6%) и гипогонадизма (у 29% мужчин и 25,3% женщин). Риск развития гипотиреоза при этом связан с применением химиолучевого лечения и более высокой СОД. Возникновение гонадотоксичности главным образом ассоциируется с влиянием алкилирующих цитостатических препаратов, используемых в большинстве схем ПХТ.

## ДОПОЛНИТЕЛЬНАЯ ИНФОРМАЦИЯ

Источники финансирования. Работа выполнена в соответствии с направлением научно-исследовательской работы НГМУ Минздрава России (тема: «Клинико-морфологические и молекулярно-биологические основы диагностики и лечения заболеваний внутренних органов и коморбидных состояний у взрослых и детей»; АААА-А15-115120910171-1), а также за счет государственного задания НИИКЭЛ — филиала ИЦиГ СО РАН по фундаментальным научным исследованиям (тема: «Изучение геномных, молекулярных и клеточных механизмов, разработка новых подходов к прогнозированию, диагностике и коррекции патологии сосудов и соединительной ткани при заболеваниях лимфатической системы, сахарном диабете и злокачественных новообразованиях»; FWNR-2022-0012).

Конфликт интересов. Авторы декларируют отсутствие явных и потенциальных конфликтов интересов, связанных с содержанием настоящей статьи.

Благодарности. Авторы выражают благодарность сотрудникам центральной исследовательской лаборатории НГМУ Минздрава России за помощь в организации и проведении исследования.

Участие авторов. Войтко М.С. — вклад автора по критерию 1 заключается в получении, анализе данных, интерпретации результатов; формировании концепции и дизайна исследования, по критерию 2 — написание статьи; Климонтов В.В. — вклад автора по критерию 1 заключается в формировании концепции и дизайна исследования, по критерию 2 — внесение в рукопись существенных правок с целью повышения научной ценности статьи; Поспелова Т.И. — вклад автора по критерию 1 заключается в формировании концепции и дизайна исследования, по критерию 2 — внесение в рукопись существенных правок с целью повышения научной ценности статьи; Шебуняева Я.Ю. — вклад автора по критерию 1 заключается в получении данных и вкладе в разработку концепции исследования; по критерию 2 — участие в написании статьи; Фазуллина О.Н. — вклад автора по критерию 1 заключается в анализе данных и интерпретации результатов; по критерию 2 — участие в написании статьи. Все авторы одобрили финальную версию статьи перед публикацией, выразили согласие нести ответственность за все аспекты работы, подразумевающую надлежащее изучение и решение вопросов, связанных с точностью или добросовестностью любой части работы.
